# Influence of propofol intravenous anesthesia on hemorheology, haemodynamics and immune function of colorectal carcinoma patients undergoing radical resection

**DOI:** 10.12669/pjms.35.3.590

**Published:** 2019

**Authors:** Jianmin Yu, Mingfen Han, Jun Geng

**Affiliations:** 1 *Jianmin Yu, Department of Anesthesiology III, Binzhou People’s Hospital, Shandong, 256610, China*; 2 *Mingfen Han, Department of Anesthesiology III, Binzhou People’s Hospital, Shandong, 256610, China*; 3 *Jun Geng, Inpatient Operating Rooms II, Binzhou People’s Hospital, Shandong, 256610, China*

**Keywords:** Haemodynamics, Hemorheology, Propofol intravenous anesthesia, Radical resection of colorectal carcinoma

## Abstract

**Objective:**

To analyze the changes of hemorheology, haemodynamics and immune function of patients during propofol intravenous anesthesia in the radical resection of colorectal carcinoma and its significance.

**Methods:**

The study included 112 patients who underwent radical resection of colorectal carcinoma in our hospital between August 2016 and December 2017, and they were divided into an observation group (N=56) and a control group (N=56) using random number table. Patients in the observation group were given propofol intravenous anesthesia, while patients in the control group received inhalation anesthesia of sevoflurane. Hemorheological and haemodynamical indexes were compared and analyzed before anesthesia (T0), 90 min after induction (T1), 150 min after induction (T2) and 30 min after entering post-anesthesia care unit (T3), and the changes of immune function before and after surgery was also observed.

**Results:**

The whole blood viscosity under high, medium and low shear rates of the observation group declined significantly compared to that of the control group at T1, T2 and T3 (P<0.05). The heart rate (HR) and systolic pressure (SPB) of the observation group significantly decreased at T2 compared to those at T1 (P<0.05), but recovered to the level observed at T0 at T3. The diastolic blood pressure (DBP) of the two groups at T1, T2 and T3 was not significantly different with that at T0 (P>0.05). The levels of CD45RA+ and CD45RO+ of both groups had a significant decrease at the end of the surgery compared to before anesthesia (P<0.05); the levels of the observation group recovered at the postoperative 72^nd^ h, and the differences with the levels before anesthesia had no statistical significance (P>0.05); the level of CD45RA+ of the control group also recovered at the postoperative 72^nd^ h, but the difference with the level before anesthesia had no statistical significance (P>0.05); the level of CD45RO+ of the control group had a significant decrease, and the difference with the level before anesthesia was statistically significant (P<0.05). The level of CD45RA+/CD45RO+ of the observation group at the end of surgery and the postoperative 72^nd^ h was not significantly different with those before anesthesia (P>0.05). The level of CD45RA+/CD45RO+ of the control group at the postoperative 72^nd^ h showed a significant increase compared to before anesthesia (P<0.05).

**Conclusion:**

Propofol intravenous anesthesia has a significant improvement effect on hemorheology before radical resection of colorectal carcinoma and has a small influence on haemodynamics. Moreover it is beneficial to the recovery of immune function. The therapy is worth promotion.

## INTRODUCTION

With the improvement of people’s living standards, dietary structure has changed, and the incidence of colorectal cancer increased year by year.^1^ Colorectal cancer is one of the common malignant tumors of the digestive tract. The early symptoms of colorectal cancer are not obvious. With the increase of tumor volume, patients will have abnormal stool, blood, diarrhea, local abdominal pain and other symptoms. It will involve the whole body in the late stage, and the mortality is very high.^2^,^3^ Surgery is considered as a radical treatment for colorectal cancer, but surgical trauma can cause non-specific physiological stress reaction, and most of patients with malignant tumors have high blood viscosity and high aggregation state.^4^ Moreover because of stress and drug use during surgery, the hemorheology and hemodynamics indexes often have abnormal changes and there are high risks of complications such as thrombosis, edema and postoperative infection.^5^ A study showed that different anesthetics used during the operation had different effects on stress response,^6^ immunosuppressive function, pain degree and complications, so the selection of anesthetics is very important to the surgical efficacy and prognosis of patients. Propofol, a kind of intravenous anesthetic, is commonly used in clinical anesthesia maintenance method. It is featured by good controllability, fast onset, short action time and fast recovery.^7^,^8^

Previous studies on propofol were mostly focused on anesthesia and analgesic effects, but less on clinical prognosis. This study was designed to investigate the effects of propofol intravenous anesthesia on hemorheology, hemodynamics and immune function in patients undergoing radical colorectal cancer surgery.

## METHODS

### General data

One hundred and twelve patients with colon or rectal cancer who underwent radical resection of colorectal cancer during August 2016 to December 2017 in our hospital were divided into an observation group and a control group using random number table method, 56 in each group. In the observation group, there were 31 males and 25 females, and they aged from 30 to 69 years old, with an average age of (54.6±7.2) years; there were 37 cases of grade I and 19 cases of grade II. In the control group, there were 30 males and 26 females, and they aged from 32 to 70 years old, with an average age of (55.2±6.3) years; there were 35 cases of grade I and 21 cases of grade II. The results were comparable as there was no significant difference in gender, age and cancer stage between the two groups (P>0.05). This study was approved by the medical ethics committee of our hospital, and all the patients signed informed consent.

### Inclusive and exclusive criteria

Patients who satisfied the diagnostic criteria of colorectal cancer formulated by Society of Oncology of Chinese Medical Association, were diagnosed as colorectal cancer by relevant laboratory examination and imaging examination and based on clinical symptoms, had surgical indications and had grade I or II American Society of Anesthesiologists (ASA) were included.

Those who underwent radio chemotherapy before surgery, had severe problems in the cardiac, hepatic and renal functions, immunity, internal secretion and circulatory system, had other malignant tumors or blood diseases, had abnormality of mentality, took anaesthetic or analgesic drugs in recent two months, was given massive transfusion during surgery, underwent a second surgery, or took anticoagulant one week before surgery were excluded.

### Therapeutic method

After admission, patients in the two groups were intramuscularly injected with 0.1 g of phenobarbital sodium and 0.3 mg of scopolamine. After entering into the surgical room, the heart rate, blood pressure and oxygen saturation were monitored, and intravenous fluid pathway was opened, electrolyte was supplied through intravenous infusion of liquid at a speed of 12~15 mL/kg·h, and indexes were detected using a GE Dash-4000 monitor.

### Patients in both groups were injected with 0.05 mg/kg of midazolam (approval number

H10980026, Jiangsu Nhwa Pharmaceutical Co., Ltd., China) and 0.3 ~ 0.4 μg/kg of sufentanil (Approval number: H20050580, Yichang Humanwell Pharmaceutical Co., Ltd., China). Then patients in the observation group were given intravenous transfusion of 1 ~ 2 mg/kg of propofol (trade name: diprivan, producer: Corden Pharma S.P.A., import drug registration number: H20130537). 0.6 mg/kg of rocuronium bromide (approval number: H20093186; Zhejiang Shijie Biopharmaceutical Co. Ltd., China) was given after the patients fell asleep and then tracheal intubation was performed. In the control group, 6 L/min oxygen flow and 8% sevoflurane (trade name: Sevoflurane, producer: Maruishi Pharmaceutical Co., Ltd., China, import drug registration number: H20090714) were used for induction firstly. The oxygen flow was adjusted to 1 L/min and the concentration of sevoflurane was adjusted to 5% after the patients fell asleep. Then 0.6 mg/kg of rocuronium bromide was intravenously transfused and trachea was intubated with tubes. As to anesthesia maintenance, patients in the control group were given constant inhalation of 2% ~ 5% sevoflurane and constant pump of 0.1 ~ 0.3 μg/kg·min. The dosage of sufentanil and propofol or sevoflurane was adjusted according to the specific conditions of patients.

### Observational indexes

Hemodynamic indexes were detected using a Succeeder SA-9000 hemorheology analyzer. The elbow venous blood was extracted before anesthesia (T0), 90 min after induction (T1), 150 min after induction (T2) and 30 min after entering post-anesthesia care unit (T3), and the whole blood viscosity under high, medium and low shear rates were detected.

Vascular hemodynamic index was monitored using a Mindray T8 electrocardiogram monitor. The heart rate (HR), systolic pressure (SBP) and diastolic blood pressure were recorded before anesthesia (T0), 90 min after induction (T1), and 150 min after induction (T2) and 30 min after entering post-anesthesia care unit (T3).

Three mL of elbow venous blood was collected from each patients before anesthesia, at the end of surgery and at the postoperative 72^nd^ h. Levels of T lymphocytes subsets including CD45RA+, CD45RO+ and CD45RA+/CD45RO+ were detected using a flow cytometry (Becton, Dickinson and Company, USA).

### Statistical analysis

SPSS 21.0 was used to process the data, and the measurement data was expressed by mean±standard deviation. The comparison between groups was performed by independent sample t-test, and the comparison between different time points was performed by ANOVA for repeated measurement. The counting data was expressed by % and compared by X^2^ test. The difference was thought statistically significant if P<0.05.

## RESULTS

### Comparison of hemorheological parameter between the two groups

The comparison of different indexes between the two groups suggested no statistical significance at T0 (P>0.05). The whole blood viscosity of patients in the observation group had a significant decreased at T1, T2 and T3 compared to that at T0 (P<0.05), and the indexes recovered slightly at T3, which was significantly different with that at T2. Compared to the control group, the whole blood viscosity of patients in the observation had a significant decrease at T1, T2 and T3 (P<0.05, Table-I).

**Table-I:** The comparison of hemorheological indexes between the two groups.

Group	Index	T0	T1	T2	T3
Observation group	Whole blood viscosity under high shear rate	5.55±1.02	4.49±0.71^ac^	4.18±0.63^ac^	4.81±0.76^abc^
	Whole blood viscosity under moderate shear rate	6.95±1.28	5.56±0.88^ac^	5.20±0.81^ac^	6.01±0.98^abc^
	Whole blood viscosity under low shear rate	14.96±2.23	12.42±1.73^ac^	11.72±1.61^ac^	13.14±1.91^abc^
Control group	Whole blood viscosity under high shear rate	6.23±0.84	5.66±0.98	5.55±1.13	6.33±1.13
	Whole blood viscosity under moderate shear rate	7.83±1.00	7.08±1.25	6.92±1.43	7.91±1.46
	Whole blood viscosity under low shear rate	16.56±1.53	15.21±2.23	14.92±2.41	16.52±2.33

***Note***: a indicated P<0.05 compared to T0, b indicated P<0.05 compared to T2, c indicated P<0.05 compared to the control group.

### The comparison of hemodynamic parameters between the two groups

There were no significant differences in different hemodynamic indexes between the two groups at T0 (P>0.05). HR in the observation group decreased significantly at T1 and T2 than at T0 (P<0.05), but increased significantly at T3 (P<0.05), and there was no significant difference between T3 and T0 (P>0.05); SBP decreased significantly at T2 compared to T0 (P<0.05) and increased significantly at T3 compared to T2 (P<0.05), and there was no significant difference between T3 and T0 (P<0.05). DBP had insignificant changes at T1, T2 and T3 compared to T0 (P>0.05). HR of the observation group was significantly lower than that of the control group at T1 and T2 (P<0.05); there was no significant difference in SBP and DBP between the two groups at T1, T2 and T3 (P>0.05, Table-II).

**Table-II:** The comparison of hemodynamic parameters between the two groups

Group	Index	T0	T1	T2	T3
Observation group	hr (times/min)	88.12±15.36	75.22±10.61^ac^	75.60±9.8^ac^	83.61±12.22^b^
	sbp (mm hg)	111.93±17.62	108.13±14.76	102.02±17.47^a^	114.02±18.13^b^
	dbp (mm hg)	78.71±14.41	80.63±8.80	78.20±12.02	81.60±12.01
Control group	hr (times/min)	87.20±13.22	85.43±10.13	86.13±10.57	86.81±12.92
	sbp (mm hg)	110.22±13.58	109.00±11.04	102.80±15.97	112.78±15.95
	dbp (mm hg)	79.02±12.38	78.78±10.08	80.01±11.22	80.75±14.02

***Note:*** a indicated P<0.05 compared to T0, b indicated P<0.05 compared to T2, indicated P<0.05 compared to the control group.

### Comparison of levels of T lymphocytes subsets between the two groups

There was no significant difference in levels of T lymphocytes subsets including CD45RA+, CD45RO+, CD45RA+/CD45RO+ between the two groups before anesthesia (P>0.05). At the end of the surgery, the levels of CD45RA+ and CD45RO+ in the two groups were significantly lower than those before anesthesia (P<0.05); the levels of CD45RA+ and CD45RO+ in the observation group recovered slightly at the postoperative 72^nd^ h after surgery and were not significantly different with those before anesthesia (P<0.05); the level of CD45RA+ in the control group recovered slightly as well at the postoperative 72^nd^ h after surgery and was not significantly different with that before anesthesia (P>0.05), and the level of CD45RO+ in the control group was significantly lower than that before anesthesia (P<0.05). The level of CD45RA+/CD45RO+ of the observation group at the end of the surgery and at the postoperative 72^nd^ h was not significantly different with that before anesthesia (P>0.05). The level of CD45RA+/CD45RO+ of the control group was significantly higher than that before anesthesia at the postoperative 72^nd^ h after surgery (P<0.05, Table-III).

**Table-III:** The comparison of the levels of T lymphocytes subsets between the two groups.

Group	Index	Before anesthesia	At the end of surgery	Postoperative 72^nd^ h
Observation group	CD45RA+(%)	44.81±7.83	41.08±6.59^a^	43.62±6.84
	CD45RO+(%)	41.76±6.31	35.33±7.12^a^	40.91±6.84^c^
	CD45RA+/CD45RO+	1.12±0.98	1.22±0.81	1.13±0.83^c^
Control group	CD45RA+(%)	43.79±6.78	40.82±5.73^a^	42.94±6.38
	CD45RO+(%)	40.86±6.39	36.33±6.96^a^	25.71±6.28^a^
	CD45RA+/CD45RO+	1.18±0.74	1.24±0.61	1.86±0.86^a^

***Note:*** a indicated P<0.05 compared to T0; c indicated P<0.05 compared to the control group.

## DISCUSSION

Early colon cancer can be cured by radical surgery^9^, but perioperative patients have changes in hemorheology and hemodynamics because of factors such as stress. Red blood cells play a major role in hemorheology. The rheological property of red blood cells is determined by their own deformability and aggregation.^10^ It mainly affects the viscosity and patency of blood in microcirculation. The changes of rheological property of red blood cells will result in the increase of the viscosity and patency of blood, decrease of microcirculation function, reduction of returned blood volume, decrease of cardiac output and insufficiency of organ perfusion, which finally leads to organ failure.^11^,^12^ In addition, the increased blood viscosity will increase the risk of thrombosis. Hemodynamics of blood in the cardiovascular system is called hemodynamics, and its main contents include blood pressure, blood volume, resistance and their interaction.^13^ The most intuitive and simple hemodynamic index in routine clinical monitoring is blood pressure.^14^

Propofol is a kind of alkylphenol derivative with strong fat solubility and one of the short-acting intravenous anesthetics commonly used in clinic. Compared with the conventional intravenous inhalational anesthesia, propofol can maintain a more stable anesthesia, improve recovery quality, and achieve a better depth of anesthesia.^15^ Although propofol anesthesia has been widely used in clinic, its effect on hemorheology is still controversial. A study suggested that propofol intravenous anesthesia might lead to microcirculation disturbance and arteriovenous thrombosis,^16^ while Fleck T et al. found that propofol could improve hemorheological parameters in elderly patients to reduce the probability of postoperative thrombosis. In this study,^17^ compared with T0, the whole blood viscosity of the observation group under high, middle and low shear rates decreased significantly at T1, T2 and T3, and were significantly lower than that of the control group. It was concluded that intravenous anesthesia with propofol could effectively reduce the hemorheological parameters, similar to previous studies.^18^,^19^

It also showed that propofol intravenous anesthesia could effectively prevent thrombosis compared with sevoflurane. In the study of hemodynamics, the HR and SPB of the observation group at T2 was significantly lower than that at T0, but there was no significant difference between the two indexes at T3 and T0; the DBP of the two groups had no statistically significant difference at different time points. It suggested that the two anesthesia methods had no excessively large effect on the hemodynamics of patients with colorectal cancer during the operation, and the drug use was safe, which is in line with the conclusion of Budic I et al.^20^

The proliferative cancer cells in patients with colorectal cancer can affect their normal physiological function, resulting in poor immune function, hence the tolerance of surgery and anesthesia is usually poor.^21^ A study has shown that anesthetics had a temporary effect on immune function,^22^ and there are still potential threats to this effect although the body may become normal after withdrawal. CD45RA+ and CD45RO+ are important T lymphocyte subsets which can regulate cellular and humoral immunity. CD45RA+ cells are primitive T cells that are not stimulated; CD45RO+ cells are functional cells that are differentiated after being stimulated by antigens; and the elevation of CD45RA+/CD45RO+indicate the deterioration of disease. The results showed that the levels of CD45RA+ and CD45RO+ in the two groups at the end of surgery were significantly lower than those before anesthesia (P<0.05); the levels of CD45RA+ and CD45RO+ in the observation group recovered slightly at the postoperative 72^nd^ hour, and there was no significant difference comparing to before anesthesia (P>0.05); the level of CD45RA+ of the control group also recovered at the postoperative 72^nd^ hour and was not significantly different with that before anesthesia (P<0.05), and there was a significant difference in CD45RO+ at the postoperative 72^nd^ hour and before anesthesia (P<0.05). The level of CD45RA+/CD45RO+ of the observation group at the end of surgery and at the postoperative 72^nd^ h was not significantly different with that before anesthesia (P>0.05). The level of CD45RA+/CD45RO+ of the control group had a significant increase at the postoperative 72^nd^ h compared to before anesthesia (P<0.05). It indicated that sevoflurane inhibited the activity of T lymphocyte subsets in patients after operation, and the effect lasted for a long time, while propofol anesthesia which was relatively stable had no significant effect on the immunity of patients. The reason might be that propofol had small irritation to the body, took effect rapidly and caused mild stress reaction.

## CONCLUSION

Intravenous propofol anesthesia can significantly improve hemorheology related indicators of colorectal cancer patients and has little effect on hemodynamics, which is conducive to the recovery of patients’ immune function. The results can provide a reference for clinical anesthesia for patients with colorectal cancer.

### Authors’ Contribution

**YJM:** Study design, data collection and analysis.

**YJM, HMF & GJ:** Manuscript preparation, drafting and revising.

**YJM:** Review and final approval of manuscript.
